# Subacute Sclerosing Panencephalitis: Results of the Canadian Paediatric Surveillance Program and review of the literature

**DOI:** 10.1186/1471-2431-5-47

**Published:** 2005-12-15

**Authors:** Craig Campbell, Simon Levin, Peter Humphreys, Wikke Walop, Renee Brannan

**Affiliations:** 1 Section of Pediatric Neurology, Department of Pediatrics, Children's Hospital of Western Ontario, University of Western Ontario, London, Canada; 2 Division of Neurology, Department of Pediatrics, Children's Hospital of Eastern Ontario, University of Ottawa, Ottawa, Canada; 3 Public Health Agency of Canada, Government of Canada, Ottawa, Canada

## Abstract

**Background:**

Subacute Sclerosing Panencephalitis (SSPE) is so rare in developed countries with measles immunization programs that national active surveillance is now needed to capture sufficient number of cases for meaningful analysis of data. Through the Canadian Paediatric Surveillance Program (CPSP), the SSPE study was able to document a national incidence and determine the epidemiology of affected Canadian children.

**Methods:**

Between 1997 and 2000, the CPSP surveyed monthly 1978 to 2294 Canadian pediatricians and sub-specialists for SSPE cases. The response rate varied from 82–86% over those years.

**Results:**

Altogether, four SSPE cases were reported to the CPSP: one case before, two during and one after the study period. The incidence of SSPE in Canadian children was 0.06/million children/year. Of the four cases, diagnosed between ages four and 17 years, three children had measles infection in infancy. All children showed a progressive course of dementia, loss of motor skills and epilepsy. Two children were treated with isoprinosine and intraventricular interferon but died in less than three years from disease onset. One child did not have any treatment and died after seven years of illness. One child received intraventricular ribavirin and remains alive, but markedly impaired, nine years following diagnosis.

**Conclusion:**

The CPSP has demonstrated that Canadian paediatricians and paediatric neurologists may encounter cases of SSPE. This report highlights the clinical course of affected Canadian children and provides a review of the disease and its management.

## Background

Measles infection and its associated complications have become uncommon in Canada since widespread immunization was implemented in 1963. One such complication, Subacute Sclerosing Panencephalitis (SSPE), is a devastating, progressive, central nervous system destructive process caused by mutant measles virus infection of neurons. Typically, affected persons have a history of measles infection in infancy period [1]. Years later the clinical features of SSPE develop. The disease is characterized by dementia, deterioration of motor function and epilepsy. Although decline may be punctuated by periods of clinical plateau, a gradual progressive course leading to death is invariable.

Because of the uncommon nature of SSPE in Canada the epidemiologic and clinical experience with the disease is limited. The Canadian Paediatric Surveillance Program (CPSP) initiated a study in 1997 [2] to determine a national incidence and the epidemiological features of this disorder, including any potential contribution that a vaccine-strain virus might have in to the etiology of this disease.

The purpose of this article is to present the four cases that were reported to the CPSP and review the literature with a focus on the clinical issues and management of the disease.

## Methods

The Canadian Paediatric Surveillance Program is a national program developed to conduct population-based identification of rare diseases through active surveillance of Canadian pediatricians and paediatric subspecialists. The SSPE proposal was approved by the CPSP Steering Committee and funded by Health Canada. All Canadian pediatricians were provided with an SSPE case definition (Table 1) and protocol [2] on initiation of the surveillance study. Between January 1997 and December 2000, the CPSP sent an initial report form monthly to an average of 2157 pediatricians and pediatric neurologists with a query as to whether or not a new case of SSPE had been encountered. If a case was identified a follow-up epidemiological questionnaire focusing on the demographic data, history of illness and diagnostic tests was completed by the reporting pediatrician. Duplicate cases were eliminated. Only non-nominal data were collected, namely sex and birth date, to maintain patient confidentiality. Updates on number of reported cases were provided quarterly. The response rate ranged between 82–86% during this time period. Given the severity of this condition, the awareness created by the widespread distribution of educational material and the high response rate, one can assume that the population under surveillance approximates the Canadian child population.

**Table 1:** Case Definition.

Definite Case
A. High titres of serum antibodies against measles virus and the presence of oligoclonal measles virus antibodies in CSF (Serum: CSF measles antibody ratio indicative of intrathecal antibody production).
And/or
B. Measles virus antigen detected in brain tissue by biopsy or at autopsy
Suspect Case
A. Typical clinical history: usually insidious onset of mental deterioration, followed (usually within a few months) by motor dysfunction, final progressive decerebration and untimely death
And
B. Typical EEG changes

The information from the case report form was supplemented by clinical information from the original reporting pediatricians when possible. In the third clinical report further details of the brain biopsy unfortunately could not be obtained. Searches of Medline using the OVID platform were conducted for treatment trials and other clinical descriptions of affected pediatric populations.

## Results

### Clinical report 1

A 6-year-old girl of Filipino origin presented to the emergency department in July 2001 with an eight-day history of progressive weakness and abnormal movements. The movements consisted of sudden head drops described by parents as brief sleeping episodes and new onset of dropping objects. She had also been noted to have a fine tremor, fidgetiness, unsteady gait and frequent falls. Her behavior was described as more irritable and emotionally labile. Parents had noted a longer history of difficulty understanding her speech, decreased recall of the names of common objects, and requiring help to feed/use washroom. Past medical history revealed measles infection at four months of age in the Philippines and that she received standard Canadian immunizations upon immigration at age two years.

On exam the child was pleasant and cooperative but was not able to comply with two step commands. She did not know her parent's first names, her phone number or address. Cranial nerve, motor and sensory exam were all normal. A fine intention tremor was evident in the upper extremities. Every 5–10 seconds the child would have a brief myoclonic movement of the whole body with head flexion, eye closure and arm extension. She would often fall during these myoclonic jerks but otherwise her gait and balance appeared intact.

A video EEG confirmed the presence of high amplitude periodic discharges [See Additional file 1] associated with head drops. Investigations from the CSF were normal except for oligoclonal banding and markedly elevated measles virus IgG at 7100 mIU/ml consistent with the diagnosis of SSPE. The diagnosis was confirmed within 3 weeks of initial presentation. A Brief Assessment Examination showed a score of 18 with 60 being the greatest impairment (see below). An initial MRI was normal but within three months a second MRI showed diffuse cerebral atrophy and a hyperintense lesion in the left temporal lobe on T2 weighted images.

Treatment with high dose valproic acid and clonazepam significantly improved the myoclonic seizures. Isoprinosine was started immediately (100 mg/kg divided qid) upon diagnosis but by one month later on admission for insertion of the Ommaya reservoir and initiation of intraventricular interferon-alpha (IFN-α) 2B her clinical state had deteriorated. She was minimally responsive, no longer vocalizing needs, had few purposeful movements and poor head control. Despite this the treatment with intraventricular IFN-α 2B was initiated at 1 million units per day for 2 weeks, then weekly for six weeks, then every other week for three months. No improvement in the child's function occurred but no further deterioration was noted. Other symptomatic treatments included g-tube feeding, physiotherapy, glycopyrrolate to decrease oral secretions. Mild thrombocytopenia and fevers with injections occurred but did not interfere with the treatment. Due to a lack of clinical improvement, the interferon and isoprinosine were discontinued after five and eighteen months respectively.

Gradually the clinical picture progressed to diffuse rigidity, brisk reflexes with clonus and multifocal myoclonus. The child remained stable albeit in a state of minimal responsiveness, with no communication or purposeful movement until she died of a respiratory illness in March 2004, two and a half years after her diagnosis. No autopsy was performed.

### Clinical report 2

A 14-year-old boy born in Canada presented in late 1997, at seven years of age, with staring spells, diagnosed as absence epilepsy but over the next year developed atonic seizures consisting of head drops, myoclonic seizures, and then generalized tonic clonic seizures lasting approximately two minutes. Approximately one year after the onset of his seizures he was losing skills such as tying his shoelaces, and dressing himself and needed to be transferred out of a regular classroom.

His past medical history was significant for being born at 26 weeks gestation, and having mild respiratory distress syndrome and then bronchopulmonary dysplasia. No intracranial pathology was detected clinically or by head ultrasound at that time. Since infancy there had been no significant illnesses. There was no history of measles disease or contact in the first year of life. His immunization record confirmed that all immunizations had been appropriately given including MMR in June 1992 at the age of 21 months and measles booster at 6 years of age.

Clinical examination revealed a head circumference of 53.7 cm (80th percentile). Cranial nerves were intact including a normal fundoscopic exam. There was mild hypotonia and normal power. Deep tendon reflexes were generally brisk. Plantars were upgoing bilaterally.

Initial EEG demonstrated generalized 3 Hz spike wave and polyspike and wave. A subsequent EEG five months later showed marked abnormality with no normal rhythmicity and intermittent projected activity consisting of high voltage delta and multifocal sharp waves in bursts with attenuation of faster frequencies. These occurred at times in a quasi periodic fashion approximately every five seconds throughout the recording. In addition there were multiple independent spikes and sharp waves. MRI showed high signal in the periventricular white matter. CBC, electrolytes, calcium, magnesium, liver function tests, renal function tests, lactate, ammonia, creatine kinase, plasma and urine amino acids, urine organic acids, very long chain fatty acids, mucopolysaccharides and oligosaccharides were all normal. Skin biopsy did not show any inclusions on electron microscopy. Toxicology screen normal. The major findings of note were in the cerebrospinal fluid obtained in April 1999. Nucleated cell count was 3 × 106/L and protein was 662 mg/L. CSF IgG was 212 mg/L (nr 0–64) and CSF IgG/albumin ratio was 1.91 (nr 00-0.23). CSF measles titre was 2460 mIU/ml (nr = 0). The diagnosis of subacute sclerosing panencephalitis was made in April 1999. Given the lack of infant measles infection, the history of prematurity and the atypical EEG the diagnosis of SSPE was only arrived at after extensive investigation into all neurodegenerative disease.

He was not treated with specific antiviral therapy. At the time of his most recent visit, age 131/2 years, he was having seizures every three weeks consisting of clonic movements of the face and both arms lasting up to one minute. He was using a wheelchair outside the home. On examination he remained very interactive socially and appropriately verbally responsive to simple questions. He was somewhat disinhibited. Cranial nerves were intact. Fundoscopic exam showed peripheral diffuse retinal atrophy consistent with previous retinal choroiditis. There was a mild right dystonic hemiparesis superimposed on a moderate spastic diplegia. In addition there was marked cerebellar tremor in the upper limbs, significant dysmetria and gait ataxia. This child recently died due to respiratory illness and no autopsy was performed.

### Clinical report 3

A 14 year old girl born in Canada presented in March 1996 at six years of age with a history of a respiratory infection two months prior to her presentation. From that time she had been lethargic, slow to complete school assignments and unable to understand simple requests around activities of daily living. Her sleep pattern had also become disturbed. One month later she began to have drop attacks. The only other symptom of note was headache for five days. Her chest x-ray demonstrated bronchopneumonia and she was treated Erythromycin at her local hospital. A CT scan and EEG were reported to be normal

Her past medical history revealed that she was the product of a normal delivery at 38 weeks, weighing 2.5 kg. At 13 months of age she contracted pneumococcal meningitis but had a complete recovery from this. Because of her meningitis, she had not received her MMR immunization. At 14 months of age she contracted measles. Subsequently she had recovered from her illnesses and her development had been entirely normal.

Clinical examination revealed a lethargic but cooperative girl. There was bilateral papilloedema and focal chorioretinitis. Cranial nerves were otherwise intact. The motor examination was entirely normal. She had a mildly asymmetric gait but there was no ataxia.

EEG showed multiple independent spikes and background slowing. Her MRI was normal. Lumbar puncture revealed a pressure of 36 mmHg but in March 1996 the CSF glucose (3.7 mmol/L) protein (195 mg/L) leukocyte count (3 × 10-6/L) were normal. CSF measles PCR was negative. Initial treatment included clonazepam, valproate and dexamethasone. Over the next two weeks she deteriorated dramatically to the point where she would give only one word answers, demonstrated marked ataxia and significant decline in cognition. Myoclonic seizures were evident. A further EEG revealed periodic complexes. Plasma measles IgG by enzyme immunoassay was 4.34 (EIA units cutoff value < 0.7). CSF measles PCR was negative. No virus was cultured but the CSF measles IgG by enzyme immunoassay was 3.58 EIA units (nr = 0).

Within one month of her presentation she had deteriorated to the point where there were periods of time when she appeared not to be aware of her surroundings and was ataxic to the point where she needed to be fully supported when walking. She had only occasional words and continuing myoclonia. She was treated with intravenous ribavirin 100 mg/kg/day as a q 6 h regimen and ribavirin levels were measured both in the plasma and CSF. It was felt that the levels obtained in the CSF were not adequate to inhibit viral replication, and therefore, it was decided to give intraventricular ribavirin using an Ommaya reservoir. The dose was gradually increased over ten days from 1 mg/kg/day to 10 mg/kg/day. This treatment did not produce any reversal in her clinical symptoms and signs and was discontinued.

At the time of her last visit, age 13 years, the major issues were that she was sleeping through most of the day, being awake for a total of 4–6 hours per day in periods of half to one hour. Her seizures had continued to be a major problem but had improved significantly on a small dose of topiramate. Clinical examination revealed no interaction and evidence of a severe spastic quadriparesis. There was evidence of decerebrate posturing.

### Clinical report 4

A 16 year old boy born and raised in Iraq until 6 years of age, presented with a diffuse red rash on his upper extremities and trunk in September 1998, followed by a rapid change in behavior over the next three months. He was described as having memory problems, social isolation and poor concentration. He then developed myoclonic jerks of the extremities and was admitted for investigation four months after his rash.

At one year of age the child had suffered a measles-like illness. Family claimed he had received immunizations upon entering Canada but this was not confirmed.

On examination the child manifested significant mental status changes. He was disoriented in all spheres and unable to read, write or speak. Formal motor and sensory exams were not able to be performed given his mental status. There were almost continuous myoclonic seizures.

EEG demonstrated quasi-periodic bursts of high voltage slow wave activity at intervals of 4–9 seconds which correlated with myoclonic jerks seen in the patient. The EEG background was otherwise normal. MRI showed focal signal abnormalities in the left occipital lobe without enhancement. Serum and CSF showed elevated levels of measles IgG (exact values not available) in January 1999. A brain biopsy did not clearly delineate findings of SSPE.

The child was treated with isoprinosine 1 gram tid from diagnosis until death. He also received, via an Ommaya reservoir, intra-venticular IFN-α 1 million units twice a week for two weeks, then three times a week for three weeks and then once a week for a month and then it was discontinued. Seizures were treated with valproic acid 750 mg tid.

He was transferred to a pediatric rehabilitation centre after his diagnosis and in October 1999 he was brought back to hospital with generalized body rigidity, fever and agitation. He developed adult respiratory distress syndrome and renal failure due to rhabdomyolysis. Creatine kinase levels were 47,000. The specific cause for his sudden deterioration was not clear with infection, malignant hyperthermia or dystonic spasm directly related to SSPE considered. He died within two weeks of re-admission and no autopsy was performed.

## Discussion

The following sections will focus on a review of the epidemiologic, clinical and treatment issues of SSPE. For the interested reader a discussion of the pathophysiology of this unusual infection is dealt with in several important references but will not be discussed in this article (3–8).

### Epidemiology

The four cases identified in this investigation highlight the rare nature of SSPE in Canada. Of the four cases presented only two were actually incident cases during the surveillance period with one case each being diagnosed just before and just after the monitoring. So, if the sampled time period is typical, then the incidence is 0.5 cases per year in Canadian children. Using Canadian Census data for the endpoint of the surveillance period (2001) for the pediatric population (under 19 years) then the incidence would be 0.06 per million children per year (2 cases per 7.778 million children per 4 years). This incidence is consistent with other post immunization countries [9]. In Canada the annual number of cases of measles infection, which is a reportable disease, in the period just preceding the CPSP study (1990–1997) ranged from 204–6178 with a median of 808. Following the introduction of a 2-dose program and catch-up campaigns in 1997 the total number of measles infections have been 259 over 4 years (1998–2001) with 199 of these cases linked to a series of outbreaks in 2000 [10] Two of the children in this study contracted measles infection in other countries. In the other two children the risk of SSPE related to measles cases in Canada can be estimated based on the probable time of their infection. Both children were born in 1990 with case #3 having measles infection in 1991. Assuming the other child's exposure to be in the first 2 years of life then estimate of SSPE can be made using these two cases and the total number of Canadian measles cases from 1990–1991. There were 1000 cases of measles in 1990 and a large out break in Canada in 1991 with 6178 cases [10], which translate into a risk of 2 cases per 7178 measles cases or 278 cases/million measles infection which is consistent with other estimates in developed countries (See Table 2.), but is higher than formerly reported [1].

**Table 2:** Epidemiologic Studies of SSPE

**Country and years (reference)**	**Study method**	**Incidence and immunization status of population**	**SSPE cases per Measles cases in general population in relevant time period (cases of SSPE/million cases of measles)**
State of California, USA, 1998–2003 [41]	Passive surveillance.	5 cases over 5.5 years. Active immunization.	4 cases related to a 2 year epidemic of 16,400 cases of measles in California. (243 cases/million)
Bulagria 1978–2002 [42],[43]	University hospital sample.	40 cases over 25 years. No population figures given. Active immunization.	Not available.
South China 1988–2002 [44]	Physician survey and hospital record diagnostic code search.	5.5/million children. Active immunization.	At least 6 cases related to a epidemic of 4140 cases in one year. (1449 cases/million)
England and Wales 1990–2002 [45]	Passive surveillance.	47 cases (not all pediatric). No incidence figures given. Active immunization.	1 case per 25,000 measles infections. 1 case per 5,500 if measles infection in first year. (40–200 cases/million)
Japan 1977–1999 [46]	Unclear from abstract.	0.58/million population	Not available.
Papua New Guinea 1997–1999 [47]	Single hospital sample.	98/million child population. Active immunization but poor coverage.	Not available.
Australia 1995–1998 [48]	Active surveillance.	0.02/100,000 children per year	Not available.
Brazil 1990–1996 [49]	Survey of child neurologists with 84% response rate.	48 cases over 7 years	Not available.
USA 1980 [1]	Passive surveillance.	0.06/million population under 20 years	8.5 cases of SSPE/million cases of measles infection for 1960–1974.

Several countries have reported on the epidemiology of SSPE through different methods of surveillance and these are presented in Table 2. Estimates of the risk of SSPE related to total population and the number of measles infections are included in the table. In some cases these estimates are derived from the total number of measles cases in a specific region during a specific epidemic. We have converted these figures to show the cases of SSPE per million cases of measles infection and note a large range from 8.5–1449 cases/million.

Only one of the cases reported in this study actually occurred in a child of Canadian origin whose infection pathogenesis was as a result of the standard exposures usually encountered by Canadian children. The other three cases were more typical in their pathogenesis, occurring following a clinically obvious measles infection in infancy. As in most described SSPE cases the measles infection in the latter three patients happened before age two years [11]. In all of the literature examined for this paper there is no evidence that vaccine virus strains were the cause of any case of SSPE even when no obvious clinical measles infection occurred.

### Clinical features

The typical presentation is that of a child presenting with cognitive deterioration combined with ataxia and/or myoclonus. Families may comment on head drops or events which may be mistaken for brief episodes of sleep or common childhood falls. The myoclonic events are frequently whole body spasms that occur in a periodic nature every 4–10 seconds. The brief body stiffening is often associated with eyelid closure and abduction of the arms but, can be subtle with a sudden head drop or buckle of the knees. The periodic myoclonus is typically quite disabling even when patients are still functioning well intellectually. Other seizure types can be encountered, as seen in case 2, but the periodic myoclonic event is characteristic of the disorder. More uncommon presenting features have been described such as: tremor, dystonia, headache and hemiparkinsonism [12], chorioretinitis [13], hallucinations [14]. The history of a measles infection may be difficult to determine, as in our first case, either due to a mild course, or to cultural or language differences if adequate translational services are not used.

The clinical features have been divided into four stages [15]. These stages are shown in Table 3. Progression through these stages is variable with a well recognized fulminant or acute course where the patient develops at least 66% neurologic disability (as measured by the NDI, see Table 3) in the first 3 months or death within six months [16]. In contrast protracted survival, in the late stages of the disease usually in a vegetative state, has been described both in natural history and treated case reports.

**Table 3:** Clinical outcome measures used in SSPE.

**Measure**	**Descriptor**
Jabbour Stages [15]	
Stage IA	Behavioral, cognitive and personality change only.
Stage IB	Myoclonic Spasms: focal and not periodic.
Stage IIA	Further mental deterioration. Myoclonic spasms: periodic, generalized, frequently causing drop spells precluding ambulation
Stage IIB	Apraxias, agnosias, language difficulties. Motor signs: spasticity, ataxia. Ambulation with assistance only.
Stage IIIA	Speaking less, visual difficulties. Sits up. Myoclonic spasms frequent (every 3–5 seconds). May have seizures.
Stage IIIB	No spontaneous speech, poor comprehension, blind. Myoclonic spasms. Bedridden, dysphagia. EEG background delta. Other abnormal movements: chorea, ballismus.
Stage IV	No myoclonic spasms. EEG low voltage with no periodic slow wave complexes. Patient in a vegetative state.
Neurologic Disability Index (NDI) [23]	
Behavioral/Mental	Irritability, Personality, Withdrawal, Intelligence and Higher cortical function
Myoclonia/Seizures	Location, Repetition, Frequency, Seizures (major), Synchrony
Motor/Sensory	Reflexes and tone, Strength and bulk, Abnormal postures and incoordination, Movements, Sensory modalities
Vegetative/Systemic	Vision, Hearing, Verbalizations, Autonomic, Nutritional
For each characteristic a score is given from 0 to 4 indicating 'No abnormality' to 'Profound abnormality'. Total Score is 80 and converted to a percentage. A higher score indicates greater impairment.	
Brief Assessment Examination (BAE). [22]	
Alertness	No response = 8. Minimum general response = 6, Specific response (35% level) = 4. Specific response (70% level) = 2. Normal = 0.
Receptive Speech/Language and Cognitive	Follows commands 0–3. Points at objects 0–3. Points at colours 0–3. Points at shapes 0–3.
Visual Digit Span	Zero digits = 8. One digit = 6. Two digits = 4. Three digits = 2. Four digits = 0.
Speech	No response= 8. Severe dysarthria = 6. Moderate dysarthria = 4. Mild dysarthria = 2. Normal = 0.
Mathematics	No skills = 8. Counts to five = 6. Counts to ten = 4. Single digit addition = 2. Single digit subtraction = 0.
Pictorial Memory	Zero objects = 8. One objects = 6. Two objects = 4. Three objects = 2. Four objects = 0.
Total Score is out of 60 with greater scores reflecting greater impairment. Modifications are outlined for children less than 6 years of age.	

The most characteristic aspect of the myoclonic events is their 4–10 second periodic nature. The episodes are associated with a high amplitude bi or triphasic spike or slow wave discharge on EEG with a normal background initially. With time the background rhythm deteriorates and the myoclonic discharges become less evident.

CT scans are rarely helpful, but may show low density changes in affected areas. MRI abnormalities are most commonly seen in the white matter with high signal intensity in T2 and FLAIR images [17]. One study focusing solely on the MRI findings in 26 patients with SSPE showed that normal scans were uncommon, seen in only 3 patients, all in the early stages of disease [18]. Isolated cortical signal change was equally uncommon in this series with only one child showing this finding. Most of the high signal lesions were in subcortical white matter or periventricular white matter, or both. Lesions in the corpus callosum were noted in only six children but never as an isolated finding. Five patients demonstrated either mass effects or contrast enhancement in association with lesions. Diffusion weighted change on MRI may be associated with the white matter lesions [19] but there is little evidence currently. Deep gray matter hyperintensities have been reported but are less common [19,20]. Progressive cerebral atrophy can be demonstrated on follow-up imaging. Uncommonly, large 'mass-like' cortical and subcortical lesions have been reported on MRI [12].

MRI spectroscopy has been carried out in six patients with SSPE and compared to healthy controls and no differences are noted in the early stages of disease. Later in the clinical course decreased N-acetylaspartate and increased choline and myo-inositol peaks did distinguish the affected children, reflecting neuronal loss, glial proliferation and inflammation respectively [21].

### Outcome measures

Efforts have been made to use traditional psychology assessments (eg. Bayley Scales) to monitor responses to treatment in SSPE but the degree of impairment and the fact that these measures were developed for developing infants not dementing children with physical limitations has led to difficulties in administration and scoring [22]. In response to these problems two measures have been developed and used in the setting of SSPE to follow change and assess response to treatment (Table 3).

The first measure, the Neurologic Disability Index (NDI) [23], may be the most sensitive to change but is poorly described and complicated to apply. The second measure, the Brief Assessment Examination (BAE), has been developed specifically for SSPE [22]. This measure has been described in detail so that it is easily reproduced. Furthermore, the BAE is weakly correlated with the NDI but more importantly highly correlated with disease stage. Data on the test-retest reliability over four time intervals was reported in the original description but the rapid clinical deterioration of most children in the sample made only short interval correlations fall into the excellent range [22].

### Treatment

The pharmacologic management of SSPE can be divided into disease modifying agents and symptomatic therapies. The latter is largely directed at seizure control and all the evidence is empirically based. Many different anticonvulsants have been used and one report of successful treatment of myoclonic movements with trihexyphenidil is on record [14]. The disease modifying agents are outlined in a table found in additional file 2 with sections divided by level of evidence rather than by drug as many combination therapies have been evaluated.

Two medications, isoprinosine (or inosiplex) and interferon, have received the most attention. The first agent is not an antiviral agent but rather an immune modulating substance that facilitates lymphocyte immune function once triggered by a viral antigen [24]. In the setting of viral infections a general immunodepressive state is encountered, both in animal models and humans, which isoprinosine has been shown to improve by promoting lymphocyte proliferation and the production of immunoglobulin and lymphokines. Interferon has received attention because it activates natural killer cells and directly inhibits viral replication [25]. Typically in SSPE IFN-α is administered intraventricularly through an Ommaya reservoir, but the use of beta-interferon subcutaneously has been reported in a case series of seven patients with clinical improvement or stabilization noted in three [26].

Until recently no randomized controlled trials of isoprinosine and interferon had been conducted. In response to this the International Consortium on SSPE conducted a single blinded, randomized controlled trial enrolling patients from 1996 until 2000 [27]. The experimental group received isoprinosine and intra-ventricular IFN-α and the control group was treated with isoprinosine alone given that a placebo control was felt unethical. Survival analysis showed no significant difference in groups over the first 6 months after which the dropout rate was so high no statistical analysis could be completed. No significant difference was noted on the BAE or the NDI over two years follow-up. Despite 121 subjects being randomized only about half this number was used in any of the outcome evaluations primarily due to lack of initial baseline assessments but also due to 19 protocol violations. No intention to treat analysis was presented.

In a case control study Anlar et al. (1997) [28] compared the clinical outcome of 22 children treated with isoprinosine and intra-ventricular IFN-α with 35 children treated with isoprinosine only. IFN was given as 100,000 units/m^2 ^increasing to 1 million units/m^2 ^per day for 5 days/week for six weeks at a time and repeated at 2–6 month intervals. Little data was given for the control group and it is not clear why they did not receive IFN-α given they were diagnosed during the same period. Half of the IFN group improved initially and until approximately eight years following diagnosis these children enjoyed a better survival profile. Although many case reports describe clinical benefits for children treated with IFN there are several case reports that report poor outcomes [29,30]. CSF measles antibody titres may fall with treatment [31] however it is not clear that this is always related to clinical improvement [26,29,32,33].

Although there does not appear to be a substantial difference to clinical outcome with the addition of IFN to isoprinosine compared to natural history data, the number of patients who stabilize on treatment suggests that isoprinosine at least is helpful. These conclusions are supported by observational research. The effectiveness of isoprinosine was demonstrated in a Japanese multi-site retrospective study of 89 children treated with isoprinosine and 62 untreated [34]. The dosage used ranged from 26–190 mg/kg/day (mean 63 mg/kg/day) and the duration was as long as five years and one month. Similar initial disease characteristics were noted between the groups but the children who received isoprinosine were three times more likely to have been additionally treated with IFN, whereas the 'untreated' group received corticosteroids more frequently, reflecting a change in clinical practice during the study period. Kaplan Meier curves demonstrated a one and five year survival of 97.6% and 86.3% in the treated group compared to 75.4% and 51.2% in the untreated group. These results were found to be statistically significant. Subgroup analysis did demonstrate an apparent detrimental effect of corticosteroids and benefit of IFN on survival. Other measures of clinical course such as Jabbour and Freeman stages and physician judgment of outcome also favoured isoprinosine use. Seventeen children had 27 side effects including hyperuricemia, nausea/vomiting, changes in liver enzymes and drop in blood cell counts, but none required discontinuation of the medication. Two other observational studies found similar results [35,36].

Other immunomodulating or antiviral medications have been examined in SSPE. A randomized trial of cimetidine (20 mg/kg/day) was conducted in 14 children (randomized 1:1) who refused, did not tolerate or were unable to obtain isoprinosine [37]. Although not statistically significant the treatment group appeared to have less deterioration on the NDI after two months than placebo controls. Another randomized trial used immune stimulators in combination with isoprinosine. Children were randomized to either thymus extract in addition to isoprinosine, or an IFN stimulator plus isoprinosine, or isoprinosine alone. The three groups were compared and it was felt by the authors that the combination treatment group had better outcome compared isoprinosine alone but no differences in outcome reached statistical significance [50]. Ribavirin has been used as adjunct therapy, in addition to intra-ventricular IFN-α, in two children age 12 and 13 years old who had had SSPE for approximately eight months [38]. Some clinical improvement was noted in both after several months on the therapy. Mixed success with IVIG [39], amantadine [40], corticosteroids [40], and acyclovir [40] have been reported in case series.

The cases we present demonstrate the controversies in management. The child who received no active antiviral or immune modulating treatment has survived for seven years whereas the two children treated with a more standard regimen of isoprinosine and IFN died quickly. Further high quality randomized trials are needed to evaluate IFN and even though isoprinosine appears to be an effective agent in SSPE, this too should be subject to a randomized trial. Ongoing trials are feasible given the number of SSPE cases in countries with endemic measles infection. Although the profound deterioration of all children with this condition makes it difficult to limit therapy, it is important for the patient, physician and the health care system to have proper data validating treatment decisions. When available, pediatric palliative care services should be accessed early in the course of SSPE.

### Limitations

The use of active surveillance to document cases of disease is limited by the interest and responses of the involved pediatricians and pediatric neurologists. However, with a response rate of 82–86%, this does not appear to be a concern. Furthermore, with its dramatic clinical course, SSPE is a disease that would almost certainly be referred for pediatric neurology consultation, be managed at least at some point in a tertiary referral centre, and its rarity would generate considerable interest, all of these factors making reporting more likely. The fact that initial report forms are sent out monthly, with quarterly follow-up, and the request that even negative responses are sent in, means that it is unlikely that cases were missed.

## Conclusion

The cases reported here demonstrate the typical clinical patterns of SSPE that physicians in Canada may encounter. This report should raise awareness of the disease and will serve to re-familiarize them with the possibility of this disorder arising in the context of normal exposures to measles virus that Canadian children experience. Unfortunately, little appears to change the usual dramatic neurological deterioration over time.

## Abbreviations

SSPE: Subacute sclerosing panencephalitis

CPSP: Canadian Pediatric Surveillance Program

MMR: measles, mumps, rubella

CSF: cerebral spinal fluid

IFN: Interferon

NDI: Neurologic Disability Index

BAE: Brief Assessment Examination

## Competing interests

The author(s) declare that they have no competing interests.

## Authors' contributions

CC and SL were responsible for the initial draft of the manuscript. PH, RB and WW contributed to revisions of the manuscript.

## Pre-publication history

The pre-publication history for this paper can be accessed here:



## Supplementary Material

Additional File 1 EEG telemetry of case 1. Demonstrating periodic nature of head drop myoclonic events corresponding to high amplitude slow waves on EEG. Click here for file

Additional File 2 Treatment for SSPE. Tabular description of evidence for treatments of SSPE. Click here for file
