# Supplementary material for: Subacute Sclerosing Panencephalitis: Results of the Canadian Paediatric Surveillance Program and review of the literature
Source: BMC Pediatr. 2005 Dec 15;5:47. doi: 10.1186/1471-2431-5-47 (PMC1343569; doi:10.1186/1471-2431-5-47)
Supplement: Additional File 2 — Treatment for SSPE. Tabular description of evidence for treatments of SSPE. [file 1471-2431-5-47-S2.doc]

# Table 4. Treatment for SSPE

| Study  Reference | Treatment Groups and Regimen | Number of Subjects | Duration of  follow up | Outcome | Comments |
| --- | --- | --- | --- | --- | --- |
| Treatments evaluated by Randomized Controlled Trials | | | | | |
| [27] Gascon, 2003 | 1. Isoprinosine 100 mg/kg/d 2. Isoprinosine plus intraventricular IFN-alpha 2b, one million U/m^2^ 2x/week | Randomized  N=121   1. N=62 2. N=59   Final groups  After dropouts:   1. N=39 2. N=28 | 6 months | No difference between the groups on any outcome measure: death, NDI, BAE or stages | 1. Large drop out (30%) 2. No ITT analysis 3. Single blinding (clinical raters) 4. Short follow up |
| [37] Anlar, 1993 | - 1. Cimetidine 20 mg/kg/d (max. 200 mg tid)   2. Placebo | 1. N=7 2. N=7 | Two months of treatment and two months after treatment | Clinical benefit seen with Cimetidine on NDI (not statistically different) | 1. Small N 2. Short follow up |

| [50] Sobczyk, 1991 | 1. IFN inducer and Isoprinosine 2. Thymus extract and Isoprinosine 3. Isoprinosine only | Unknown | 6 months | Combined treatments appeared better than Isoprinosine alone (not statistically significant) | Not reviewed fully as only published in Polish |
| --- | --- | --- | --- | --- | --- |
| Treatment evaluated by Observational Studies | | | | | |
| [51] Aydin, 2003 | 1. Isoprinosine 100 mg/kg/day, plus Lamivudine 10 mg/kg/day, plus subcutaneous IFN-α 2a 10million U/m^2^ three times/week 2. Natural history controls | 1. N=19 2. N=13 | 6 months | Treatment group had lower mortality, longer survival, higher remission rate | 1. No reason given why controls not able to have treatment |

| [28] Anlar, 1997 | 1. Intraventricular   IFN- α, 1million U/m^2^/day for 5 days/ week for six weeks. Courses repeated every 2-6 months up to 6 times, plus Isoprinosine.  2. Controls treated with Isoprinosine | 1. N=22 2. N=35 | | 1. Longest follow up of 108 months. 2. Longest follow up 110 months | | Percentage of those alive higher in treated group by about 30% until 8 years of follow up then equal. | 1. Little information on control group.  2.Not clear why controls could not receive IFN. |
| --- | --- | --- | --- | --- | --- | --- | --- |
| [35] Yalaz, 1992 | 1. IFN- α and Isoprinosine 2. Natural history controls | 1. N=22 2. N=77 | | 2-54 months | | Improved scores on the NDI in 50%. Remission rate higher than controls. |  |
| [34] Fukuyama, 1987 | 1. Isoprinosine 26-190 mg/kg/day (median 63). 2. Controls (may be taking other treatments). | 1. N=89 2. N=62 | | Groups followed beyond 10 years. | | 1. Significant difference in survival on analysis of survival curves favoring Isoprinosine.  2. Proportion of mild disease higher in Isoprinosine group. | 1.Retrospective review.  2.Treated group older and diagnosed later.  3. Treated group more often on IFN. |
| [36] Jones, 1982 | 1. Isoprinosone 100 mg/kg/day 2. Historical controls from 3 different SSPE registries | 1. N=98 2. N=590 | | Life table comparisons made up to 132 months of follow up | | Survival significantly better at all intervals in the Isoprinosine group | 1. Historical controls from differing populations 2. No information on treatments in control group |
| Treatments evaluated by case report or series | | | | | | | Similar references |
| [52] Tomoda, 2003 | Intraventricular Ribavirin 1-3 mg/day for 5 days. Total course 2 months. Plus intraventricular IFN. | N=10 | Unclear | | 1. Decreased CSF measles antibiodies. 2. Clinical improvement in 6/10.   3. Side effects minimal. | |  |
| [38] Tomoda, 2001 | Intravenous Ribavirin 30mg/kg/day x 7 days then 50-70 mg/kg/day for three months. Exact interval of Ribavirin treatment not clear. Plus intraventricular IFN. | N=2 | 5 and 13 months | | Mild improvement and no deterioration. Mild side effects. | |  |
| [40] Bobele, 1999 | Amantadine for 21 days | N=1 | 7 months | | No benefit | |  |
| [26] Anlar, 1998 | Subcutaneous Beta-IFN 1a 3 million U 2-3 times/week or IFN 1b 8 million U 2x/week for at least six months. Isoprinosine as well | N=7 | N=7 (all children not able to take intraventricular IFN) | | 4-24 months  2/7 rapid death and 5/7 stable. | |  |
| [53] Cianchetti, 1998 | Intraventricular IFN- α 2a  2.2 million U/m^2^ twice a week | N=8 | 12-105 months | | Treatment effective Side effects: acquired motor neuron disease | | [15] effective  [30] not effective  [31] effective  [32] effective  [33] not effective  [54] effective |
| [39] Gurer, 1996 | IVIG 400 mg/kg/day for 5 days once a month for two months | 1 patient | 18 months | | Child clinically improved and remained stable. | |  |
| [23] Dyken, 1982 | Isoprinosine 100 mg/kg/day | N=15 | Longest 158 months | | 1. 4/15 showed continued deterioration and rapid death. 2. 10/11 survivors showed some clinical improvement. | | [55] |

Note: Randomized controlled trials identified through a Medline search looking for all randomized controlled trials in SSPE. Other trials identified by an English language, clinical trials search using text word subacute sclerosing panencephalitis.
